# Physical activity amount is associated with better attention and less daytime sleepiness in older adults

**DOI:** 10.1093/geroni/igaf122.2266

**Published:** 2025-12-31

**Authors:** Pin-Shiuan Lee, Yi-Ling Chen, Wan-Ju Cheng

**Affiliations:** National Health Research Institutes, Zhunan Town, Miaoli, Taiwan (Republic of China); National Health Research Institutes, Zhunan Town, Miaoli, Taiwan (Republic of China); National Health Research Institutes, Zhunan Town, Miaoli, Taiwan (Republic of China)

## Abstract

The natural aging process and inadequate physical activity represent modifiable and non-modifiable risk factors for cardiometabolic conditions, dementia progression, and sleep pathologies. Increased physical activities may reduce the risk of cognitive deterioration and dementia progression. While the association between sleep disorders and cognitive impairment has been established, limited research exists on the relationship between exercise, cognition, and sleep in older adults. This study recruited 147 community-dwelling older adults (62 males: 70.4 ± 6.2 years; 85 females: 69.0 ± 5.6 years) and evaluated their physical activity and sleep patterns using actigraphy. Sleep patterns were measured using Pittsburgh Sleep Quality Index and Epworth Sleepiness Scale and cognitive function was measured by psychomotor vigilance tests. Melatonin level was determined by saliva samples. Circadian rhythm measurements were analyzed by cosinor analysis, generalized additive model, interdaily stability (IS), and intradaily variability (IV). Objective sleep and physical activity pattern (power law exponent alpha, beta coefficient of a linear regression line in log-log space (GGIR), the average activity during the least active 5-h period (L5) and the most active 10-h period (M10), relative amplitude (RA), weekly vigorous, moderate, and sedentary activity, and lag 1440 mins) were retrieved from 14-days actigraphy recordings of activity counts. Results showed that participants with high-amount physical activities manifested higher IS, IV, RA, and 24-h autoregression correlation. Higher relative amplitude is associated with fewer lapses. This study reveals the positive impact of physical exercise on cognitive function and sleep, demonstrating that physical activity amount is associated with individual cognitive function and sleep qualities.

